# Simple Physical Model Unravels Influences of Chemokine on Shape Deformation and Migration of Human Hematopoietic Stem Cells

**DOI:** 10.1038/s41598-018-28750-x

**Published:** 2018-07-13

**Authors:** Takao Ohta, Cornelia Monzel, Alexandra S. Becker, Anthony D. Ho, Motomu Tanaka

**Affiliations:** 1 Department of Physics, The University of Tokyo, Tokyo, 113-0033 Japan; 2 Toyota Physical and Chemical Research Institute, Nagakute, Aichi 480-1192 Japan; 3 Center for Integrative Medicine and Physics, Institute for Advanced Studies, Kyoto University, 606-8501 Kyoto, Japan; 4 Physical Chemistry of Biosystems, Institute of Physical Chemistry, Heidelberg University, D69210 Heidelberg, Germany; 5 Department of Medicine V, Heidelberg University, D69120 Heidelberg, Germany; 6 Present Address: Experimental Medical Physics, Heinrich-Heine University Düsseldorf, 40225 Düsseldorf, Germany

## Abstract

We studied the dynamic behavior of human hematopoietic stem cells (HSC) on the *in vitro* model of bone marrow surfaces in the absence and presence of chemokine (SDF1*α*). The deformation and migration of cells were investigated by varying the chemokine concentration and surface density of ligand molecules. Since HSC used in this study were primary cells extracted from the human umbilical cord blood, it is not possible to introduce molecular reporter systems before or during the live cell imaging. To account for the experimental observations, we propose a simple and general theoretical model for cell crawling. In contrast to other theoretical models reported previously, our model focuses on the nonlinear coupling between shape deformation and translational motion and is free from any molecular-level process. Therefore, it is ideally suited for the comparison with our experimental results. We have demonstrated that the results in the absence of SDF1*α* were well recapitulated by the linear model, while the nonlinear model is necessary to reproduce the elongated migration observed in the presence of SDF1*α*. The combination of the simple theoretical model and the label-free, live cell observations of human primary cells opens a large potential to numerically identify the differential effects of extrinsic factors such as chemokines, growth factors, and clinical drugs on dynamic phenotypes of primary cells.

## Introduction

The balance between self-renewal and differentiation of somatic stem cells is regulated by their microenvironment (called stem cell “niche”). For example, the dormancy of the most primitive hematopoietic stem cells (HSC) is maintained by adhesion to and interaction with the bone marrow niche^1–3^. Interactions of stem cells with the marrow niche actually play crucial roles in blood cancers. In acute myeloid leukemia, leukemia initiating cells remain dormant in the marrow niche and thus can hardly be eliminated by chemotherapy^4–6^. HSC-niche interactions are regulated by a chemokine, stromal cell-derived factor 1*α* (SDF1*α*), secreted in the bone marrow, which is specifically identified by CXCR4 protein expressed on HSC^7–10^.

To date, several clinical drugs interfering with SDF1*α*-CXCR4 interactions have been approved for cancer treatment. However, the exact mode of function of such drugs (antagonist, agonist, or inhibitor) compared to naturally occurring SDF1*α* still remains controversial, because the drugs might harm the function of HSC through off-target effects. Therefore, it is highly important to develop a novel tool to quantitatively assess the influence of chemokines and drugs on human HSC functions beyond the commonly used cell phenotypes.

Recently, we fabricated the surrogate niche model surface based on planar lipid membranes displaying precisely defined concentrations of ligand molecules SDF1*α* or N-cadherin^11^. By means of a self-developed force measurement assay, we have quantitatively discriminated the adhesion strength of healthy HSC from that of leukemia blasts in the presence and absence of soluble SDF1*α*. The power spectrum analysis of stochastic morphological dynamics in Fourier space further unraveled that the energy dissipation of HSC by oscillatory deformation is strongly damped by the presence of physiological level of soluble SDF1*α* (5 ng/mL). This enables one to quantitatively assess and compare the influence of SDF1*α* and drugs on the “dynamic phenotypes” of HSC, which is not accessible by commonly used image analysis platforms in real space.

To date, several theories have been developed to model cell dynamics and the underlying mechanisms. For example, Levine *et al*.^12^ and Sawai *et al*.^13^ have employed the so-called phase field to represent the motion of cell boundaries induced by the chemical reactions inside a cell. However, these models could be applied only for cell lines genetically expressing reporter molecules, but not for primary human subjects. Moreover, these models did not explicitly consider the adhesion between the cell and substrate, although the adhesion-induced contraction forces drive cell migration. Along this line, the two-dimensional model of Ziebert and Aranson introduced the degrees of freedom for adhesion^14^, and Tjhung *et al*. have generalized the theory of active polar fluids to study crawling of a three-dimensional cell^15^. But, the effects of extrinsic factors on adhesion and migration have not been investigated. Theories of active gel have also been utilized as one-dimensional models of motile cells, but they are currently not able to handle the shape deformation^16,17^. Last but not least, it should be noted that all the models mentioned above are expressed by a complicated set of partial differential equations, which involves fairly heavy numerical computations. Therefore, the quantitative comparison of these models with the data generated from primary human samples is still not practically possible.

Ohta *et al*. recently proposed a simple physical model of crawling cells that includes the frictional effect between substrates and cells into the time-evolution equations^18^. This model, represented by ordinary differential equations, enables us to qualitatively reproduce both a stationary motion of fish keratinocyte driven by time-independent deformation forces and a non-stationary motion of *Dictyostelium discoideum* driven by time-dependent, excitable forces.

In the present study, we extended this strategy to model the deformation and migration of primary human HSC in the absence and presence of extrinsic SDF1*α*. The frictional coupling between HSC and the surrogate surfaces can be controlled precisely by the self-assembly of adhesion ligands. To investigate the non-stationary dynamics (active deformation and migration) of HSC, our new mathematical model introduces the frictional coupling and oscillatory internal forces. By sharply focusing on deformation and migration, which are accessible from the label-free, live cell images, our models can be quantitatively compared to the experimental results. This enabled us to numerically represent the effect of chemokine SDF-1*α* as the nonlinear coupling in the equation of motion, which distinctly alters the persistence of migration trajectories. Such an interdisciplinary combination of dynamic phenotypes of cells and theoretical models opens new avenue to discriminate differential functions of clinical drugs compared to that of natural chemokine.

## Model of Crawling Cells

In this section, we describe our model for cell crawling. A migrating cell on a substrate is approximated as a two-dimensional object. Deformation around a circular shape is represented as

1 $$R(\theta ,t)={R}_{0}(1+\delta R(\theta ,t)),$$

where *R*_0_ is the radius without deformation and *θ* is the angle from the *x*-axis. The deviation *δR*(*θ*, *t*) can be expanded in a Fourier series as

2 $$\delta R(\theta ,t)=\sum _{n=-\infty }^{\infty }{c}_{n}(t){e}^{in\theta }.$$

Since uniform expansion and contraction of a circular cell are prohibited and a translational motion of the cell is represented by the migration velocity of center of mass **v** = (*v*_1_, *v*_2_), the modes *c*_0_ and *c*_±1_ should be removed from the Fourier series (2). We write the migration velocity as *v*_1_ = *v* cos *ζ* and *v*_2_ = *v* sin *ζ*. Similarly the Fourier components of deformations are set as

3 $${c}_{n}=\frac{{s}_{n}}{2}{e}^{in{\theta }_{n}},$$

with the real amplitude *s*_*n*_ and the real phase *θ*_*n*_.

The details of our model for cell crawling are described in Appendix. The basic time-evolution equations for a migrating cell are given by^18^

4 $$v=\mathrm{2|}\gamma |{s}_{2}\,{s}_{3},$$

5 $$\zeta =3{\theta }_{3}-2{\theta }_{2}-{{\rm{\Psi }}}_{v},$$

and

6 $$\frac{d{s}_{2}}{dt}=-\,{\kappa }_{2}{s}_{2}+\frac{{b}_{0}{v}^{2}}{2}\,\cos \,(6{\theta }_{23}+2{{\rm{\Psi }}}_{v})+\,{g}^{(2)}(t)+{\xi }_{2}(t),$$

7 $$\frac{d{\theta }_{2}}{dt}=-\,\frac{{b}_{0}{v}^{2}}{4{s}_{2}}\,\sin \,(6{\theta }_{23}+2{{\rm{\Psi }}}_{v})+\,\frac{{g}^{(2)}(t)}{{s}_{2}}{\eta }_{2}(t),$$

8 $$\frac{d{s}_{3}}{dt}=-\,{\kappa }_{3}{s}_{3}+\frac{{d}_{0}{v}^{3}}{4}\,\cos \,(6{\theta }_{23}+3{{\rm{\Psi }}}_{v})+\,{g}^{(3)}(t)+{\xi }_{3}(t),$$

9 $$\frac{d{\theta }_{3}}{dt}=-\,\frac{{d}_{0}{v}^{3}}{12{s}_{3}}\,\sin \,(6{\theta }_{23}+3{{\rm{\Psi }}}_{v})+\,\frac{{g}^{(3)}(t)}{{s}_{3}}{\eta }_{3}(t),$$

where *θ*_23_ = *θ*_2_ − *θ*_3_. Equation (4) implies that the cell can migrate only when both deformation modes *s*_2_ and *s*_3_ exist. Therefore, the present model is a model of deformation-induced migration. The proportional constant |*γ*| is the mobility which characterizes the degree of friction between the cell and substrate. The constant phase difference Ψ_*v*_ in eq. (5) is fixed as Ψ_*v*_ = *π* throughout the present paper. This means that if *θ*_2_ = *θ*_3_ = 0 the cell with two convex parts in the front and one convex part in the rear (i.e., Y-shaped cell) migrates to the left along the *x* axis. The time-evolution eqs (6–9) consist of four parts. The shape relaxation occurs with the relaxation rates *κ*_2_ and *κ*_3_. In the numerical simulations given below, we set *κ*_3_ = 2*κ*_2_ to reduce the free parameters. The nonlinear coupling between deformation and migration is expressed by the terms with the coefficients *b*_0_ and *d*_0_. We assume that these constants are positive so that the cell elongates along the direction of the migrating velocity^18^. The deformation forces acting on the *n*-th deformation mode are denoted by *g*^(*n*)^(*t*) whose form is given shortly below. The other time-dependent terms *ξ*_*n*_ and *η*_*n*_ are the random forces acting on the amplitude and the angle of deformations, respectively. We assume that these are not time-correlated and distributed uniformly in the interval $-{\epsilon }_{g} < {\xi }_{n} < {\epsilon }_{g}$ and $-\epsilon  < {\eta }_{n} < \epsilon$. The constants ${\epsilon }_{g}$ and $\epsilon$ are fixed as $\epsilon =0.2$ and ${\epsilon }_{g}=0.025$ throughout the present paper.

In our previous paper^18^, the forces are generated by the so-called coherence resonance to represent a stochastic and excitable property in a consistent manner with the experiments on *Dictyostelium* cells^13^. On the other hand, our previous experimental results^11^ suggest that human hematopoietic stem cells predominantly undergo periodic deformation. Therefore, in this study, we introduced oscillatory deformation forces, such as:

10 $${g}^{(2)}(t)={g}_{c}^{(2)}+{g}_{0}^{(2)}{(\frac{1+\cos (\omega t)}{2})}^{2},$$

11 $${g}^{(3)}(t)={g}_{c}^{(3)}+{g}_{0}^{(3)}{(\frac{1+\cos (\omega t+{\varphi }_{g})}{2})}^{2},$$

with the frequency *ω* and the phase difference *ϕ*_*g*_. In the numerical computations given below, we choose these quantities as *ω* = 2*π*/10, *ϕ*_*g*_ = −*π*/4. Note that the period *T* = 2*π*/*ω* = 10 corresponds to 5 min which remains in the same order of magnitude of the observed cell deformation frequency in the present experiments^11^. The constants ${g}_{c}^{(n)}$ and ${g}_{0}^{(n)}$ (*n* = 2, 3) are positive. To reduce the number of the free parameters, we put the relations among these constants as ${g}_{c}^{(2)}={g}_{c}^{(3)}={g}_{c}$, ${g}_{0}^{(2)}=2{g}_{c}$, and ${g}_{0}^{(3)}={g}_{c}$.

From the solutions of eqs (4–9), the location and the shape of the cell at each time are determined as

12 $$x(t)={x}_{cm}(t)+r(t)\,\cos \,(\alpha )$$

13 $$y(t)={y}_{cm}(t)+r(t)\,\sin \,(\alpha ),$$

with

14 $$r(t)={r}_{0}\mathrm{[1}+{s}_{2}\,\cos \,2(\alpha -{\theta }_{2})+\,{s}_{3}\,\cos \,3(\alpha -{\theta }_{3})],$$

where *α* changes from 0 to 2*π* and *x*_*cm*_ and *y*_*cm*_ denote the location of the center of mass of the cell.

We make several remarks about the model given by Eqs (4–9). This is probably one of the simplest model systems showing that deformation of a cell induces its migration. It was derived solely by symmetry argument. The product of the symmetric second and third rank tensors produces a vector. This relation between the deformation and translational velocity causes both elongation and head-tail asymmetry of a cell and seems to hold generally for crawling cells. We have introduced the internal forces to change the cell shape. When the force is constant, the model produces migration with an elongated constant shape. As described explicitly in ref.^18^ when *b*_0_, *d*_0_ > 0, the elongation of a cell is parallel to the migration direction whereas when *b*_0_, *d*_0_ < 0, the elongation is perpendicular to the migration direction as observed in keratocyte cells. The former case was considered in the present study. To express an oscillation of cell shape, we have employed the time-dependent forces given by Eqs (10, 11). It is mentioned here that this kind of active force is necessary for coarse-grained models in terms of a few modes of deformation as in the case of amoeboid swimming^19^. Our model does not include microscopic processes inside cells, such as biochemical reactions and signal transduction. We will show in the following section that this simplicity of the model is an advantageous point to compare quantitatively with the experiments of HSC since it is not possible to introduce molecular reporter systems into primary cells from human donors.

## Results and Discussion

### Analysis of Migration and Deformation

**Figure 1:**
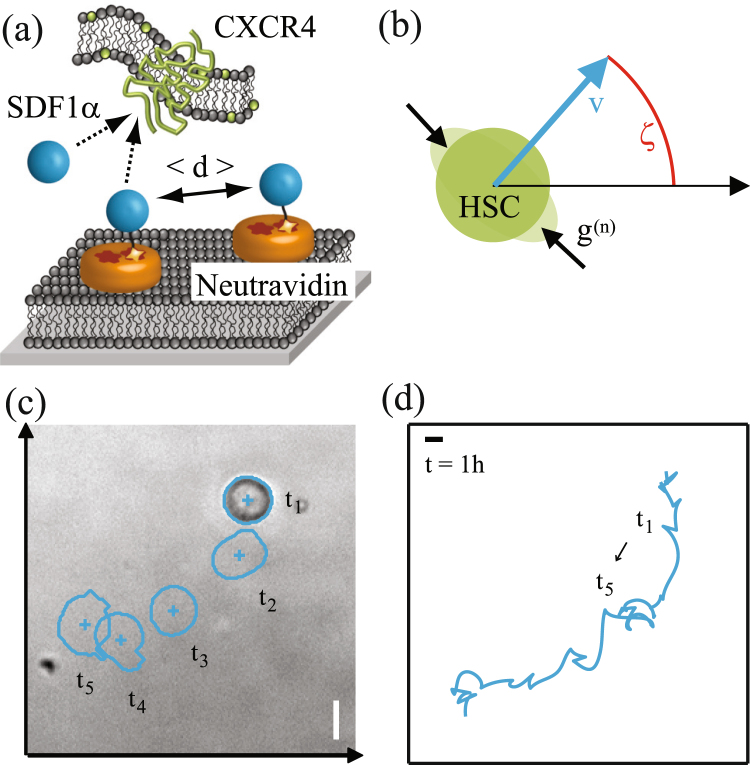
Schematic representation of (**a**) experimental system and (**b**) theoretical variables where *g*^(*n*)^ stands for the *n*-th deformation force. The angle between the migration velocity and the *x* axis is given by *ζ*. (**c**) Tracking of cell center and periphery from the phase contrast images. (**d**) Cell trajectory for 1 h. Scale bar: 10 *μ*m.

Figure 1 displays the schematic illustrations of (a) the experimental system and (b) the theoretical model. The superposed snapshots of a migrating human HSC and the trajectory are depicted in panels (c) and (d), respectively. The center of mass was extracted from each frame of live phase contrast images captured at *t* = *t*_1_, *t*_2_, *t*_3_… as in Fig. 1(c). The trajectory was recorded over 1 h with the time interval of 40 s.

**Figure 2:**
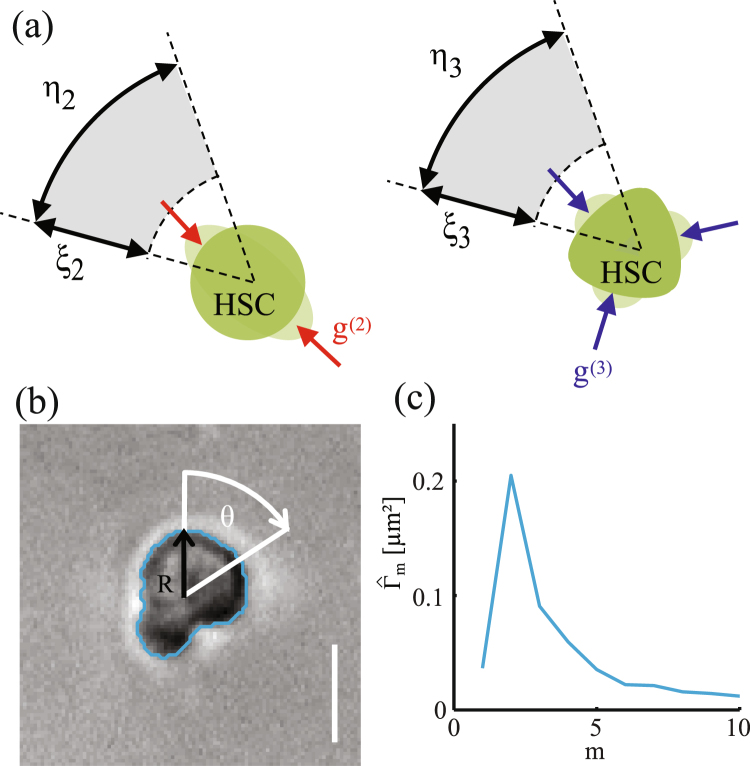
(**a**) Deformation forces *g*^(*n*)^, and noises *ξ*_*n*_ and *η*_*n*_ (*n* = 2, 3) acting on the amplitude and the direction of the force, respectively. (**b**) Parameterization of deformation amplitude *R*(*θ*, *t*), and (**c**) Power spectrum of cell deformation ${\hat{{\rm{\Gamma }}}}_{m}$. Scale bar: 10 *μ*m.

Figure 2 shows (a) the forces acting on the cell in our model, and (b) and (c) the experimental analysis of cell deformation. Characteristic spatio-temporal patterns from stochastic dynamics of HSC was extracted as follows^11,20,21^. First, the peripheral edge of the cells was defined from the phase contrast time-lapse images, and then the radial distance *R* between the center of mass and periphery was plotted in polar coordinate, *R*(*θ*, *t*) as in Fig. 2(b). From these data, the autocorrelation function was calculated as

15 $${{\rm{\Gamma }}}_{rr}({\rm{\Delta }}\theta ,{\rm{\Delta }}t)=\frac{\langle R(\theta +{\rm{\Delta }}\theta ,t+{\rm{\Delta }}t)R(\theta ,t)\rangle }{\langle R{(\theta ,t)}^{2}\rangle }.$$

The autocorrelation maps of HSC exhibited much poorer features compared to the autocorrelation function maps of cancer cells^21^, because HSC is a compact cell whose interior is mostly filled with cell nucleus. In this study, we analyzed the power spectrum from the Fourier transformation of shape deviation $${R}_{m}(t)=(1/2\pi )$$$${\int }_{0}^{2\pi }d\theta R(\theta ,t)\,\exp \,(im\theta )$$ ^22,23^:

16 $${\hat{{\rm{\Gamma }}}}_{m}=\langle {R}_{m}(t){R}_{-m}(t)\rangle .$$

Note that the isotropic expansion/contraction (*m* = 0) and the translational motion (*m* = 1) are not assessed, since we took the center of mass as the origin of inertial frame. Here, the cell deformation is originated from active processes that is driven by energy consumption, such as bending of cell membranes and remodeling of cytoskeletons^24,25^. Thus, the mode analysis of power spectra enables one to identify the predominant mode of deformation that HSC dissipates the energy. The results are displayed in Fig. 2(c).

### Migration and Deformation in the Absence of Chemokine

**Figure 3:**
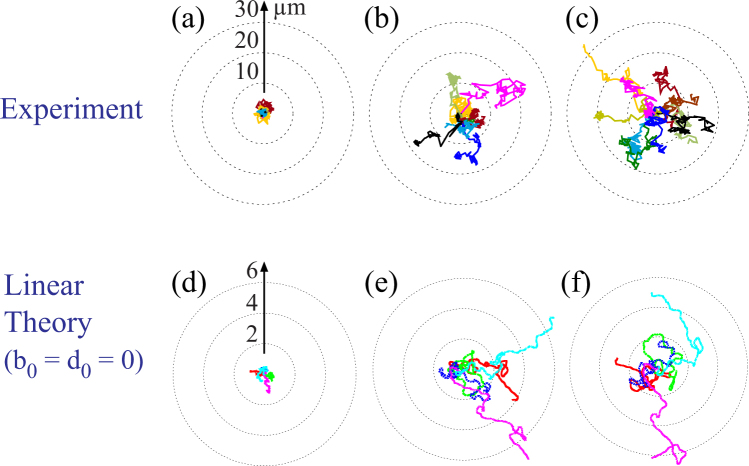
Migration trajectories of HSC on membrane displaying SDF1*α* at 〈*d*〉 = (**a**) 6 nm, (**b**) 18 nm, and (**c**) 34 nm for 1 h. The corresponding theoretical trajectories obtained from five independent runs for the linear case (*b*_0_ = *d*_0_ = 0) are shown in panels (**d**–**f**), respectively. The values of the parameters in the simulations are listed in Table 1. The radius of the three concentric circles is 2, 4 and 6 in the dimensionless unit. These corresponds to 10 *μ*m, 20 *μ*m and 30 *μ*m in the experimental trajectories. The data of the trajectories for 880 < *t* < 1000 are plotted.

Figure 3(a–c) represents the experimentally traced migration trajectories of HSC on model niche surfaces displaying SDF1*α* at the average intermolecular distance between the neighboring neutravidin molecules 〈*d*〉 = 6, 18, and 34 nm, respectively. Each line coincides with a trajectory monitored for 1 h. These three conditions were selected based on our previous account, where we quantified the adhesion strength of HSC to the membrane-based bone marrow niche model displaying SDF1*α* by using microinterferometry and the self-built pressure wave assay^11^. Using the pure phospholipid membranes as the control, the transition from strong to weak adhesion was found at 〈*d*〉 = 10–15 nm. Thus, for simplicity, we define substrates with 〈*d*〉 = 6 nm as “sticky”, 〈*d*〉 = 18 nm as “intermediate” and 〈*d*〉 = 34 nm as “sloppy” for the comparison with the model. When the niche surface was “sticky”, (〈*d*〉 = 6 nm), almost no translocation could be observed, suggesting that cells undergo mainly a random spinning motion (Fig. 3(a)). In case of an “intermediately sticky (intermediate)” surface (〈*d*〉 = 18 nm), some trajectories showed a distinct elongation (Fig. 3(b)). When the surface became “sloppy” (〈*d*〉 = 34 nm), the cells seemed to be unpinned from their initial positions, exhibiting more stretched migration trajectories (Fig. 3(c)). The magnitude of shape deformation for each value of 〈*d*〉 will be discussed later in the section “Quantitative Comparison of Experiments and Simulations”.

Now we carry out numerical simulations of our model to compare with the experiments. Among the three free parameters *γ*, *κ*_2_ and *g*_*c*_ in our model, the mobility |*γ*| is of most importance since it should be directly related to the experimentally accessible quantity 〈*d*〉. Since the relation between these two quantities is not known experimentally, we set two conditions that are necessary from the physical point of view. One is that the mobility is proportional to the free area on the substrate, i.e., |*γ*| ∝ 〈*d*〉^2^ − *a*^2^ where *a* is the diameter of the neutravidin molecules, *a* = 5.2 nm^26^. The other is that, for large limit of 〈*d*〉, the mobility should approach a finite value otherwise the migration velocity becomes infinitely large. Therefore, we approximate the relation between |*γ*| and 〈*d*〉 by the interpolation formula for 〈*d*〉 > *a*

17 $$|\gamma |=\frac{{\langle d\rangle }^{2}-{a}^{2}}{{\sigma }_{0}+{\sigma }_{1}\langle d\rangle +{\sigma }_{2}{\langle d\rangle }^{2}},$$

where the constants *σ*_0_, *σ*_1_ and *σ*_2_ are to be determined.

In the present theory, we employ the space and time units such that *R*_0_ = 1 and the relaxation rate of the second deformation mode *κ*_2_ = 1 in the intermediate surfaces. Since the cell diameter is about 10 *μ*m, we have the correspondence 5 *μ*m = 1 for the length unit. The relaxation rate of deformations is not available experimentally. Here we assume the correspondence that 0.5 min = 1 for the time unit. For example, the time duration of the experimental trajectories in Fig. 3 is 1 h which means 120 in the theory. We will see shortly below that this correspondence gives us a quantitative coincidence in comparison between the theory and experiments.

Figure 3(d–f) represent theoretical trajectories of the migration of cells calculated from five independent runs for a linear case (*b*_0_ = *d*_0_ = 0), corresponding to the “sticky”, “intermediate”, and “sloppy” surfaces, respectively. Parameters used in the linear case are listed in Table 1. We have changed the parameters; the friction constant 1/|*γ*|, the relaxation rates *κ*_2_, and the magnitude of the deformation force *g*_*c*_. All of them decrease by decreasing the degree of stickiness. This is required from the physical point of view since the cell is softer and the deformation force is weaker for large 〈*d*〉. The values of the parameters such as |*γ*| = 2, 5, and 7 in Table 1 have been chosen such that the simulation results agree with experiments as satisfactorily as possible. By the correspondence between |*γ*| = 2, 5, and 7, and 〈*d*〉 = 6, 18, 34 nm, respectively, the coefficients in eq. (17) are determined as *σ*_0_/*a*^2^ = −0.594, *σ*_1_/*a* = 0.585 and *σ*_2_ = 0.640 × 10^−1^. It is notable that the theoretical model seems to well explain the qualitative tendency suggested by the experimental data: the increase in 〈*d*〉 enables the cells to explore the wider region. On a sticky surface (Fig. 3(d)), the cell is strongly pinned on the surface, showing no remarkable translocation of its center of mass. This was attributed to a random rotating motion of a poorly deformable cell^11^. On an intermediate surface (Fig. 3(e)), the cell started translational movement driven by the deformation in *m* = 2 and 3. Finally, on a sloppy surface (Fig. 3(f)), the trajectories are further expanded. Therefore, the theoretical behavior in Fig. 3(d–f) is qualitatively consistent with the experimental observations in Fig. 3(a–c).

**Table 1:** Parameters in the linear case *b*_0_ = *d*_0_ = 0.

	|*γ*|	*κ*_2_	*g*_*c*_
sticky	2.0	1.5	0.12
interm.	5.0	1.0	0.1
sloppy	7.0	0.8	0.07

The values on the lines of sticky, interm., and sloppy were used to obtain Fig. 3(d–f), respectively.

### Influence of Chemokine on Deformation and Migration

**Figure 4:**
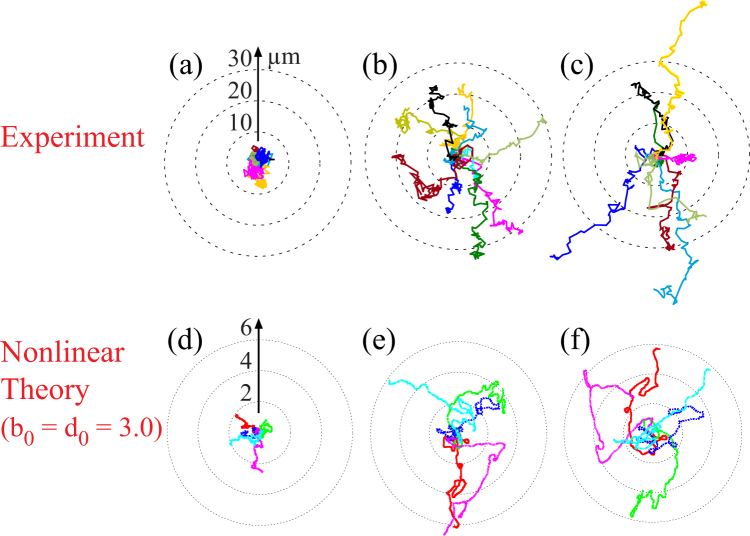
Influence of soluble chemokine SDF1*α* on migration trajectories of HSC. The experimental results measured on surrogate surfaces displaying membrane-anchored SDF1*α* at 〈*d*〉 = (**a**) 6 nm, (**b**) 18 nm, and (**c**) 34 nm for 1 h. The corresponding theoretical trajectories obtained from five independent runs for the non-linear case (*b*_0_ = *d*_0_ = 3.0) are shown in panels (**d**–**f**), respectively. The values of the parameters in the simulations are listed in Table 2. Others are the same as those in the caption of Fig. 3.

In the next step, we investigated how chemokine in solution (SDF1*α*) influences the deformation and migrational motion of HSC. Figure 4(a–c) represents the migration trajectories of HSC on surrogate surfaces functionalized with SDF1*α* at 〈*d*〉 = 6, 18, and 34 nm, respectively. Different from the results presented in Fig. 3(a–c), the experiments were performed in the presence of soluble SDF1*α* (5 ng/mL) in the medium, which is relevant to the physiological level in bone marrow. At 〈*d*〉 = 6 nm (Fig. 4(a)), HSC exhibited no clear sign of translational motion, undergoing a localized random motion. The area in which HSC moves seems slightly larger than what we found in the absence of SDF1*α* (Fig. 3(a)). The increase in 〈*d*〉 to 18 nm (Fig. 4(b)) and 34 nm (Fig. 4(c)) leads to the stretching of trajectories. Compared to the corresponding data in the absence of SDF1*α* in solutions (Fig. 3(b,c)), HSC traveled over much larger areas. In fact, the start-to-end distance of some trajectories at 〈*d*〉 = 34 nm (Fig. 4c) exceeded 40 *μ*m. Though SDF1*α* in the medium acts as a competitor to membrane-anchored SDF1*α*, our finding cannot be explained only by the decrease in adhesion area due to the competitive binding. In fact, the power spectrum analysis indicated that the magnitude of deformation is significantly damped by the presence of soluble SDF1*α*^11^, suggesting that not only the frictional coupling but also the deformation is affected by the presence of soluble chemokine.

Figure 4(d–f) represent theoretical trajectories of the migration of cells calculated from five independent runs for “sticky”, “intermediate” and “sloppy” surfaces, respectively where the nonlinear coupling constants were put as *b*_0_ = *d*_0_ = 3.0. The parameters used in this nonlinear case are summarized in Table 2. As in the linear case, we have made the frictional coupling (∝ 1/|*γ*|), the relaxation rates and the magnitude of the deformation forces decrease by decreasing the degree of the stickiness. The coefficients in eq. (17) are chosen as *σ*_0_/*a*^2^ = −0.295, *σ*_1_/*a* = 0.273 and *σ*_2_ = 0.679 × 10^−1^. Note that the nonlinear effects account for the stretch of trajectories. In fact, comparing the corresponding simulations (e.g. Figs 3(e) and 4(e)), it is evident that the trajectories for the nonlinear case are more persistent. It is also found that the trajectories in the sloppy case in Fig. 4(f) are expanded, on average, compared to those of the intermediate case in Fig. 4(e) consistently with the experimental trajectories shown in Fig. 4(b,c).

**Table 2:** Parameters in the nonlinear case *b*_0_ = *d*_0_ = 3.0.

	|*γ*|	*κ*_2_	*g*_*c*_
sticky	3.0	1.2	0.1
interm.	7.5	1.0	0.08
sloppy	9.5	0.9	0.065

The values on the lines of sticky, interm., and sloppy were used to obtain Fig. 4(d–f), respectively.

### Quantitative Comparison of Experiments and Simulations

The unique advantage of our model over commonly used mathematical ones is the capability to quantitatively compare the experimental and theoretical quantities. One of the parameters changed experimentally is the average distance 〈*d*〉. In our physical model, the corresponding quantity is the mobility of cells on frictional surfaces. We have introduced the relation between these two as eq. (17).

**Figure 5:**
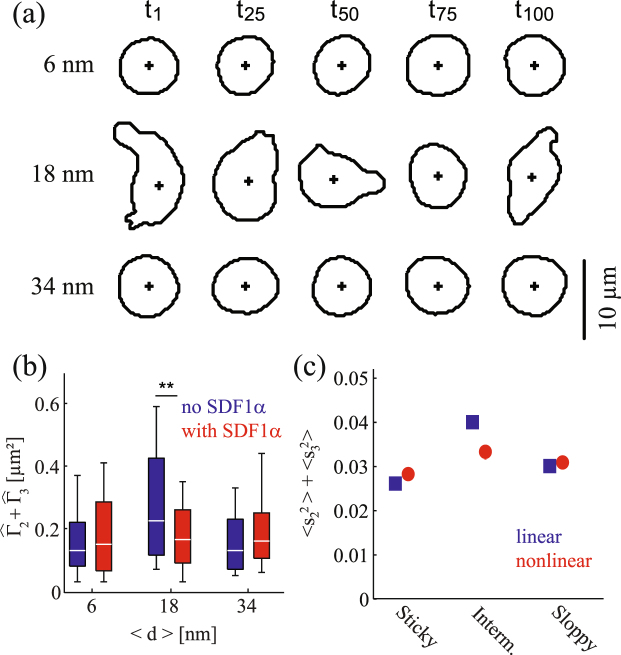
(**a**) Snapshots of a migrating cell in the absence of soluble SDF1*α* for three different values of 〈*d*〉. The suffix indicates time, e.g., *t*_100_ = 100 s. Comparison of (**b**) the sum of experimentally determined powers ${\hat{{\rm{\Gamma }}}}_{2}+{\hat{{\rm{\Gamma }}}}_{3}$ and (**c**) the sum of deformability $\langle {s}_{2}^{2}\rangle +\langle {s}_{3}^{2}\rangle$ obtained theoretically. Blue: in the absence of soluble SDF1*α* (linear case), red: in the presence of soluble SDF1*α* (nonlinear case). The error bars in the theoretical plot are smaller than the size of the symbols. The experimental values of deformations are normalized as eq. (16) including the higher modes, but the theoretical values are not. Thus, the direct comparison of the scale of the vertical axis is not possible.

The next important experimental data useful to determine the coefficients in the equation of motion are the magnitude of two principal modes of deformation; *m* = 2 and 3. Figure 5(a) displays the snapshots of a migrating cell in the absence of soluble SDF1$\alpha$ for three different values of 〈*d*〉. Figure 5(b) depicts the sum of deformability ${\hat{{\rm{\Gamma }}}}_{2}+{\hat{{\rm{\Gamma }}}}_{3}$ calculated from the power spectra as Eq. (16). One notes from Eqs (1, 3 and 16) that *s*_*n*_ = 2*R*_*n*_/*R*_0_ and therefore that ${\hat{{\rm{\Gamma }}}}_{2}+{\hat{{\rm{\Gamma }}}}_{3}=0.2$ for *R*_0_ = 5 *μ*m corresponds to ${s}_{2}^{2}+{s}_{3}^{2}=0.032$. This indicates that the theoretical result in Fig. 5(c) reproduces the correct order of magnitude for the cell deformation observed experimentally (Fig. 5(b)). Now we make more detailed comparison as described below.

The results obtained in the absence of soluble SDF1*α* are labeled in blue, while those obtained in the presence of SDF1*α* in the medium are in red. The results suggested the presence of soluble chemokine SDF1*α* does not cause a significant change in the magnitudes of deformation at 〈*d*〉 = 6 and 34 nm. However, at 〈*d*〉 = 18 nm, the magnitude of deformation in the absence of chemokine was distinctly larger than the corresponding value in the presence of chemokine. As presented in Fig. 5(c), this tendency was very well represented in our theoretical calculations, too. The deformation parametrized as ${\rm{\Sigma }}=\langle {s}_{2}^{2}\rangle +\langle {s}_{3}^{2}\rangle$ exhibits maximum for “intermediate” substrates in the linear case (Fig. 5(c) blue) consistently with the panel (a). Note that the deformation in the linear case is determined by the combination of the relaxation rate and the deformation force and is estimated as *s*_*n*_ ~ *g*_*c*_/*κ*_*n*_. Such a simple estimation of the deformations is not possible for the nonlinear case. Nevertheless, the behavior in Fig. 5(c) is consistent with the experiments in Fig. 5(b). Thus, our calculations capture the significance of active deformation and the accompanied energy dissipation deduced from the power spectrum analysis.

**Figure 6:**
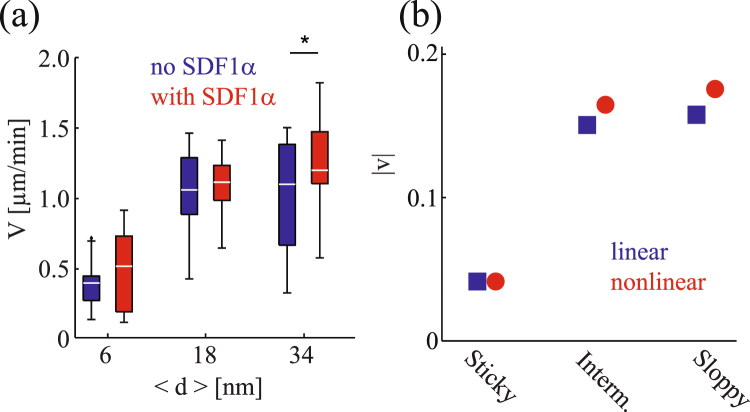
Comparison of (**a**) migration velocity *V* determined from the experiments and (**b**) |*v*| obtained from three independent simulation runs. Blue: in the absence of soluble SDF1*α* (linear case), red: in the presence of soluble SDF1*α* (nonlinear case). Note that 1 *μ*m/min in (**a**) corresponds to the dimensionless velocity 0.1 in (**b**). The error bars in the theoretical plot are smaller than the size of the symbols.

When we look at the migration speed, the velocity determined from experiments increases by increasing 〈*d*〉 and thus decreasing |*γ*| both in the absence (blue) and presence (red) of soluble SDF1*α* (Fig. 6(a)). Figure 6(b) represents the time-averaged absolute velocity |*v*| calculated from three independent simulation runs for linear (blue) and nonlinear (red) cases. First of all, both experiments and simulations indicate that the migration velocity is not significantly altered by the presence of soluble SDF1*α*. The simulation suggests that the presence of soluble SDF1*α* could result in a slight increase in the migration velocity for “intermediate” and “sloppy” surfaces, while the experiments indicate that a statistically relevant difference could be identified only from the data at 〈*d*〉 = 34 nm. Note that 1 *μ*m/min in the experiments corresponds to the theoretical velocity 0.1 in the dimensionless unit. Therefore, Fig. 6 indicates that a quantitative comparison is possible between the experiments and theory.

From the experimental data of the deformations ${\hat{{\rm{\Gamma }}}}_{2}$ and ${\hat{{\rm{\Gamma }}}}_{3}$, and migration velocity, one can estimate the mobility |*γ*| from Eq. (4). Table 3 shows the values of the mobility obtained in this way, compared to the theoretically chosen values. As presented in the table, the estimated values of the dimensionless mobility show the same tendency as the theoretical ones within experimental uncertainties, indicating that the equation of motion (Eq.(4)) properly represents the migration of HSC.

**Table 3:** Mobility |*γ*| evaluated from the experimental data of the deformations and migrating velocity in the absence and presence of SDF1*α*.

〈*d*〉 (nm)	6	18	34
no SDF1*α* ex.	4.08 ± 2.01	3.83 ± 2.41	9.13 ± 3.80
no SDF1*α* th.	2	5	7
with SDF1*α* ex.	2.59 ± 1.53	6.38 ± 3.03	7.10 ± 3.30
with SDF1*α* th.	3	7.5	9.5

The theoretical values are also given for comparison. The dimensionless values were obtained by the relation *s*_*n*_ = 2*R*_*n*_/*R*_0_ with *R*_0_ = 5 *μ*m and the correspondence of the unity of dimensionless velocity to 10 *μ*m/min.

**Figure 7:**
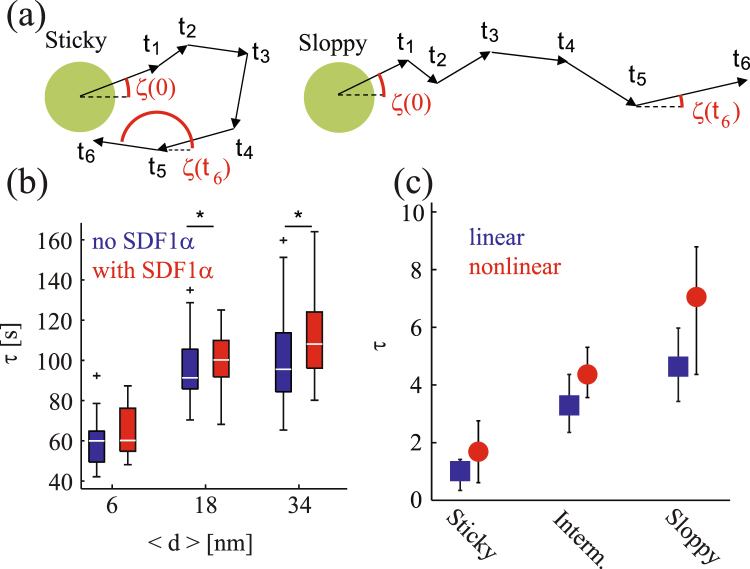
(**a**) Schematic illustration of the trajectory of a cell on a sticky and sloppy substrate. Persistence time of migration *τ* (**b**) determined from experiments and (**c**) obtained from the numerical simulations of 36 independent runs. The bars indicate the scatter of the data. Note that 90 sec in (**b**) corresponds to the dimensionless time 3 in (**c**). The meaning of the colors is the same as that in Fig. 6.

**Figure 8:**
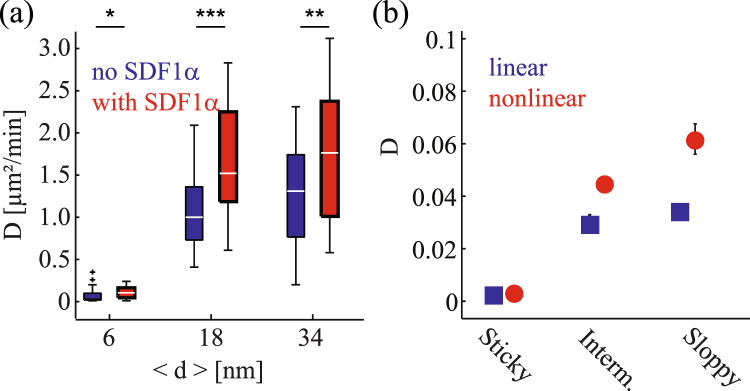
Lateral diffusion constant *D* of migrating cells determined from (**a**) the experimental results and (**b**) numerical results averaged over 36 independent simulations. Note that 1 *μ*m/min in (**a**) corresponds to the dimensionless diffusion constant 0.02 in (**b**). The meaning of the colors is the same as that in Fig. 6.

Compared to the migration trajectories in the absence of soluble SDF1*α* at 〈*d*〉 = 18 nm (Fig. 3(b)) and 34 nm (Fig. 3(c)), the corresponding trajectories in the presence of soluble SDF1*α* (Fig. 4(b,c) respectively) were clearly stretched. Consistently, the theory also showed that the trajectories for the nonlinear case are more persistent. To quantify this behavior, we evaluated the persistence time *τ* (Fig. 7) and diffusion constant *D* (Fig. 8) of a migrating cell both experimentally and numerically. The persistence time *τ* is defined through the relation

18 $$C(t)\equiv \langle \cos \,\zeta (0)\,\cos \,\zeta (t)+\,\sin \,\zeta (0)\,\sin \,\zeta (t)\rangle =\exp \,(\,-\,t/\tau ),$$

where the time is shifted such that *t* = 880 is the time origin in the theoretical plots.

Figure 7 displays (a) schematic illustration of the trajectory for a sticky and sloppy conditions, (b) the experimentally determined persistence time *τ* and (c) the corresponding value obtained by averaging the data of 36 independent simulation runs. As presented in Fig. 7(b), the experimental results show that the persistence time *τ* increases as the increase in 〈*d*〉. Moreover, the experimentally determined persistence time in the presence of soluble SDF1*α* (red) was longer than that obtained in the absence of soluble SDF1*α* (blue). This tendency was consistent with the numerical simulations where the persistence time in the nonlinear case is larger than that in the linear case (Fig. 7(c)). Here, the period of the oscillatory forces was set to be 2*π*/*ω* = 10, which is larger than the persistence time. Note that this period corresponds to 5 min in the experimental unit, which is in accordance with the characteristic period of deformations observed experimentally^11^. A small discrepancy between the experiments and theory is that the theoretical value for the nonlinear sloppy case is fairly large (though within numerical uncertainty) compared to the experimental one for 〈*d*〉 = 34 nm in the presence of SDF1*α* (red).

Figure 8 represents the lateral diffusion constant *D* of migrating cells defined through the relation

19 $$\langle {(\overrightarrow{r}(t)-\overrightarrow{r}(0))}^{2}\rangle =4Dt.$$

Figure 8(a) shows the experimentally determined diffusion constants in the absence (blue) and presence (red) of soluble SDF1*α*. On the sticky surface (〈*d*〉 = 6 nm), the diffusion constant was found to be very small irrespective of the presence or absence of soluble SDF1*α* due to the strong pinning. A prominent difference in diffusion constants was found on the intermediate surface (〈*d*〉 = 18 nm), which becomes less pronounced on the surface with 〈*d*〉 = 34 nm. Figure 8(b) shows the diffusion constant evaluated from numerical data. As in the persistence time in Fig. 7, the diffusion constant for the sloppy case is slightly larger than the corresponding experimental value for 〈*d*〉 = 34 nm in Fig. 8(a) (red). However, the behavior for the sticky and intermediate substrates is consistent with the experimental observation.

It should be noted that the experimental values of the persistence time in Fig. 7 and the diffusion constant in Fig. 8 as well as the migrating velocity in Fig. 6 are comparable with the theoretical ones quantitatively since 30 s and 1 *μ*m^2^/min correspond to 1 and 0.02 in the theoretical unit, respectively confirming the excellent agreement between experimental data and simulation results both in space and in time.

Last but not least, we remark that other possibilities of the increased persistence of migrating trajectories are unlikely in the present model. Since one of the major roles of chemokines is to serve as a chemoattractant to induce cell migrations, it is physically plausible to consider that SDF1*α* increases the noise level in eqs (6–9). However, this increase of noise intensity makes the trajectories not extended but more compact. To account for the extension of migration trajectories in the presence of SDF1*α*, there are two possibilities. One is to increase the strength of the active force, while the other is to increase the nonlinear coupling term. However the former can be ruled out because it is contradictory to the experimental results (5), implying that the deformation power in the presence of SDF1*α* is lower than that in the absence.

We have found that SDF1*α* increases the strength of the nonlinear coupling between deformation and migration to reproduce the experimental observations of human HSC. As it is expected that other chemical and biochemical cues would result in different dynamic cell behaviors, such a combination of experiments and theory opens a large potential to discriminate different effects caused by clinically used agents like pathway inhibitors. In fact, a number of studies suggested that pathway inhibitors do not only block the target molecular interaction but also interfere with other cellular functions. Thus, further systematic investigations using different extrinsic factors will enable us to confirm the conclusion mentioned above and to unravel the correlation between specific pathway activities and deformation-migration patterns (dynamic phenotypes) of primary cells, which cannot be obtained from the commonly used analysis of static cellular phenotypes. In our recent account^23^, we demonstrated that the dynamic phenotyping of deformation and migration following the same strategy can discriminate the impact of clinical HSC mobilization agents on the adhesion and migration of human HSC.

## Conclusions

In this study, we have proposed a new physical model representing the periodic deformation and migration of cells crawling in the microenvironment. Our minimal model is ideally suited for the quantitative comparison to the experiments with primary cells influenced by chemokine, which is in contrast to previously proposed models involving biochemical processes that is not accessible without molecular reporters.

As the experimental system, we chose the active deformation and migration of human hematopoietic stem cells (HSC) from umbrical cord blood on the bone marrow model surfaces, on which the frictional coupling between cells and surfaces can be controlled quantitatively. This enables one to quantitatively analyze the active deformation and migration of human HSC on substrates with various stickiness. Here, we shed light on the influence of soluble chemokine SDF1*α* that dictates the migration of human HSC in the bone marrow.

The unique advantage of this study is the direct, quantitative comparison between the experimental findings and the simulation results. We demonstrated that the linear model can explain how the adhesiveness of the substrate modulates the migration trajectories of HSC obtained from the experiments in the absence of SDF1*α* (Fig. 3). On the other hand, the nonlinear model can only recapitulate the increase in the persistence of migration trajectories observed in the presence of soluble SDF1*α*. Thus, our minimal model implies that the presence of SDF1*α* enhances the nonlinear interactions between the shape deformation and the migration velocity.

There have been several studies modeling the migration behaviors of cells under starved and vegetative conditions by the stochastic model equation for the center of mass or polarity vector^27,28^. In contrast to these studies, our model deals with not only the migration behavior but also the degree of deformations. The sum of powers for *m* = 2 and *m* = 3 deformation, reflecting the significance of energy consumed by HSC, obtained from the mode analysis of experimental power spectra ${\hat{{\rm{\Gamma }}}}_{2}+{\hat{{\rm{\Gamma }}}}_{3}$ are comparable to $\langle {s}_{2}^{2}\rangle +\langle {s}_{3}^{2}\rangle$ in simulations. The systematic comparison of other observables, such as migration velocity, persistence time of migration, and diffusion constants further confirms the quantitative agreement between experimental data and simulation results both in space and time. The combination of quantitative experiments under *in vitro* stem cell microenvironments and numerical simulations proposed here has a large potential to quantitatively identify how clinical agents and environmental parameters influence the dynamic phenotype of human primary cells.

To conclude, we have shown that the simple physical model enables us to discriminate, with a suitable choice of the parameters, different modes of motility for complex human primary cells obtained by the accurate *in vitro* experiments.

## Methods

### Preparation of membrane-based surrogate substrates

1-stearoyl-2-oleoyl-*sn*-glycero-3-phosphocholine (SOPC) and 1,2-dioleoyl- *sn*-glycero-phospho-ethanolamine-3-N-(cap biotinyl) (biotin-cap-DOPE) were purchased from Avanti Polar Lipids (Alabaster, USA), and neutravidin cross-linker from Life Technologies. SDF1*α* with and without biotin tags were purchased from R&D Systems Inc. (Wiesbaden, Germany). Iscove’s Modified Dulbecco’s Media from Life Technologies (Darmstadt, Germany) was used for all cell experiments. Glass slides were sonicated in acetone, ethanol, methanol and water for 3 min, then immersed in 1:1:5 (v/v/v) H_2_O_2_ (30%)/NH_4_OH (25%)/H_2_O and sonicated at room temperature for 3 min. The samples were kept in the same solution for another 30 min at 60 °C and rinsed with ultrapure water. Bottomless *μ*-Slide VI (Ibidi, Martinsried, Germany) were bonded onto cover slips (Gerhard Menzel GmbH, Braunschweig, Germany) using SYLGARD184 (Dow Corning Co., USA). The mixture of SOPC and biotin-DOPE were dried and suspended in buffer (150 mM NaCl, 10 mM Hepes, pH 7.5), followed by sonication for 30 min. The suspension of small unilamellar vesicles (SUVs) was injected into the chamber, incubated for 60 min at 40 °C, and the unbound vesicles were removed by rinsing with HBS buffer. The average lateral distance between lipid anchors 〈*d*〉 and thus proteins can be estimated from the molar fraction c of lipid anchors by inserting the value of the lipid area of *A*_*lipid*_ ~ 65 *Å*^2^, $\langle d\rangle =\sqrt{{A}_{lipid}/c}$. To functionalize supported membranes, the samples were incubated with neutravidin solution (40 *μ*g/mL) for 2 h, and unbound neutravidin were removed by rinsing. Then, biotinylated SDF1*α* solution (10 *μ*g/mL) was added. After removing the unbound SDF1α, the samples were equilibrated at 37 °C.

### Isolation of human HSC

All primary cells were from voluntary donors after obtaining informed consents following the guidelines approved by the Ethics Committee on the Use of Human Subjects, Heidelberg University. Human HSC, defined as CD34^+^ cells in this study, were obtained from the umbilical cord blood^11^. Mononuclear cells (MNCs) were isolated by density-gradient centrifugation (Merck KGaA, Darmstadt, Germany), and CD34^+^ cells enriched by magnetic beads were further sorted (2×) by using an AutoMACS affinity column (all Miltenyi Biotec GmbH, Bergisch-Gladbach, Germany). Non-viable cells were removed by propidium iodide staining. The final flow cytometry analysis confirmed the purity of CD34^+^ cells is higher than 95%. Each data point presented was collected from 30–50 cells from 3 donors, and the representable trajectories were shown in each polar plots.

### Live-cell tracking

A Keyence BZ-9000 (Keyence, Osaka, Japan) equipped under controlled humidity and temperature was used for live imaging of HSC migration. For each experimental condition, we collected phase contrast images from 1–2 positions, using a Plan Fluor air objective (40 ×/0.6) over 6 h (frame rate: 25 mHz). All the data were analyzed using self-written routines in Matlab 7.7.0 (R2008b) and ImageJ.

### Details of Model Equations

The shape of a deformed cell is characterized by the deformation tensors which are given in terms of the Fourier coefficients by^29^

20 $${S}_{11}={c}_{2}+{c}_{-2},$$

21 $${S}_{12}={S}_{21}=i({c}_{2}-{c}_{-2}),$$

22 $${S}_{22}=-\,{S}_{11},$$

23 $${U}_{111}=-\,{U}_{122}=-\,{U}_{212}=-\,{U}_{221}\equiv {W}_{+},$$

24 $${U}_{222}=-\,{U}_{112}=-\,{U}_{121}=-\,{U}_{211}\equiv -\,{W}_{-},$$

where

25 $${W}_{+}={c}_{3}+{c}_{-3},$$

26 $${W}_{-}=i({c}_{3}-{c}_{-3}\mathrm{).}$$

The symmetric traceless tensor *S*_*ij*_ represents an elliptical deformation and the third rank tensor *U*_*ijk*_ expresses the front-rear asymmetry. Since the higher modes of deformation seem to be less relevant in HSC (See Fig. 2(c) and ref.^11^), we consider, as a minimal nontrivial model, only the modes of *m* = 2 and 3.

By symmetry consideration, we obtain the following set of equations for *S*_*ij*_ and *U*_*ijk*_ as well as the migration velocity *v*_*i*_ of the center of mass^18^

27 $${v}_{k}=\gamma {S}_{ij}{U}_{ijk},$$

28 $$\frac{d{S}_{ij}}{dt}=-\,{\kappa }_{2}{S}_{ij}+{b}_{0}({v}_{i}{v}_{j}-\frac{{\delta }_{ij}}{2}{v}_{k}{v}_{k})+{F}_{ij}^{\mathrm{(2)}}(t),$$

29 $$\frac{d{U}_{ijk}}{dt}=-\,{\kappa }_{3}{U}_{ijk}+{d}_{0}[{v}_{i}{v}_{j}{v}_{k}-\frac{{v}_{n}{v}_{n}}{4}({\delta }_{ij}{v}_{k}+{\delta }_{jk}{v}_{i}+{\delta }_{ki}{v}_{j})]+{F}_{ijk}^{\mathrm{(3)}}(t),$$

where the repeated indices imply summation. The forces ${F}_{ij}^{\mathrm{(2)}}(t)$ and ${F}_{ij}^{\mathrm{(3)}}(t)$ contain both deterministic part and stochastic part. The coefficient |*γ*| in eq. (27) is the mobility of a cell. The relaxation rates *κ*_2_ and *κ*_3_ are positive and the interaction strengths between deformations and migration velocity are denoted by *b*_0_ and *d*_0_ in eqs (28 and 29), respectively.

We have retained in eqs (27–29) simplest nontrivial nonlinear couplings among the migration velocity and the deformation tensors. Equation (27) indicates that the cell can migrate only when both elliptical deformation and the front-rear asymmetry exist. In other words, we may identify *S*_*ij*_*U*_*ijk*_ as the polarization vector *P*_*k*_ of the deformed cell. As mentioned above, the effects of interaction between the migration velocity and the deformations are taken into account in eqs (28) and (29). These terms are necessary to distinguish deformations either parallel or perpendicular to the migration velocity depending on the sign of the coefficients *b*_0_ and *d*_0_^29^.
